# Cytokine expression during early and late phase of acute *Puumala *hantavirus infection

**DOI:** 10.1186/1471-2172-12-65

**Published:** 2011-11-16

**Authors:** Mahmoud Sadeghi, Isabella Eckerle, Volker Daniel, Ulrich Burkhardt, Gerhard Opelz, Paul Schnitzler

**Affiliations:** 1 Department of Transplantation Immunology, University of Heidelberg, Im Neuenheimer Feld 305, 69120 Heidelberg, Germany; 2 Department of Nephrology, University of Heidelberg, Im Neuenheimer Feld 162, 69120 Heidelberg, Germany; 3 Department of Infectious Diseases, Virology, University of Heidelberg, Im Neuenheimer Feld 324, 69120 Heidelberg, Germany; 4 Current address: Institute of Virology, Sigmund Freud Str. 25, University of Bonn Medical Centre, 53127 Bonn, Germany

## Abstract

**Background:**

Hantaviruses of the family *Bunyaviridae *are emerging zoonotic pathogens which cause hemorrhagic fever with renal syndrome (HFRS) in the Old World and hantavirus pulmonary syndrome (HPS) in the New World. An immune-mediated pathogenesis is discussed for both syndromes. The aim of our study was to investigate cytokine expression during the course of acute *Puumala *hantavirus infection.

**Results:**

We retrospectively studied 64 patients hospitalised with acute *Puumala *hantavirus infection in 2010 during a hantavirus epidemic in Germany. Hantavirus infection was confirmed by positive anti-hantavirus IgG/IgM. Cytokine expression of IL-2, IL-5, IL-6, IL-8, IL-10, IFN-γ, TNF-α and TGF-β1 was analysed by ELISA during the early and late phase of acute hantavirus infection (average 6 and 12 days after onset of symptoms, respectively). A detailed description of the demographic and clinical presentation of severe hantavirus infection requiring hospitalization during the 2010 hantavirus epidemic in Germany is given. Acute hantavirus infection was characterized by significantly elevated levels of IL-2, IL-6, IL-8, TGF-β1 and TNF-α in both early and late phase compared to healthy controls. From early to late phase of disease, IL-6, IL-10 and TNF-α significantly decreased whereas TGF-β1 levels increased. Disease severity characterized by elevated creatinine and low platelet counts was correlated with high pro-inflammatory IL-6 and TNF-α but low immunosuppressive TGF-β1 levels and *vice versa *.

**Conclusion:**

High expression of cytokines activating T-lymphocytes, monocytes and macrophages in the early phase of disease supports the hypothesis of an immune-mediated pathogenesis. In the late phase of disease, immunosuppressive TGF-β1 level increase significantly. We suggest that delayed induction of a protective immune mechanism to downregulate a massive early pro-inflammatory immune response might contribute to the pathologies characteristic of human hantavirus infection.

## Background

Hantaviruses of the family *Bunyaviridae *are emerging zoonotic pathogens which cause two distinct syndromes in humans: hemorrhagic fever with renal syndrome (HFRS) and hantavirus pulmonary syndrome (HPS) [1-3]. Immunopathogenesis of both syndromes is characterized by increased vascular permeability resulting in severe capillary leakage and hemorrhagic diathesis. It is therefore suggested that these similar underlying pathogenic mechanisms are at least partly immune-mediated. Hantaviruses are transmitted via rodents which are asymptomatically infected and spread the virus to humans by aerosolized secretion such as urine and feces [4]. Typical modes of exposure are occupational forest work, cleaning activities in contaminated buildings and outdoor activities in areas were bank voles are abundant. In 2010, a hantavirus epidemic was observed in Germany with more than 2000 notified cases of hantavirus infections which is the highest number ever since mandatory reporting started [5]. Southwest Germany is the main endemic area within Germany for infection with *Puumala *virus (PUUV) which causes a mild form of HFRS, called nephropathia epidemica (NE). Although NE is generally a mild disease with an incubation time of usually 2-3 weeks, after initially influenza-like symptoms, acute renal failure with anuria or oliguria, proteinuria, hematuria and thrombocytopenia is typical is seen [6-8]. Clinical severity of NE varies considerably, but prognosis is good and mortality is low [1].

Most clinical studies indicate an important role of pro-inflammatory cytokines in the immunopathogenesis of HFRS/HPS [9-11]. The efficient anti-hantaviral cell-mediated immune response in patients is mainly due to the generation of cytotoxic CD8+ T-lymphocytes early in the course of disease [12,13]. Important target cells of hantavirus in humans are monocytes and macrophages, which may also play an important role in the systemic spread of hantavirus from the primary site of infection, as well as endothelial cells. Endothelial cells, monocytes and macrophages as well as platelets can be a rich source of cytokines/chemokines during the infection with hantavirus and contribute to the HFRS/HPS immunopathogenesis [9-12]. Although most data are from patients with HFRS/HPS, immunohistochemical staining and gene polymorphism studies showed an association of pro-inflammatory cytokines and disease in PUUV infection with significantly elevated serum levels of TNF-α, IL-6, IL-2 and IFN-γ in blood and urine [2,14,15].

Two recent studies shed further light on the immunopathogenesis of acute PUUV infection [13,16]. Recently, Saksida et al reported increased levels of IL-10, INF-γ and TNF-α in both Dobrava (DOBV) and PUUV infected patients in Slovenia [16]. The authors found a significant correlation of IL-10 and TNF-α with a more severe course of disease in DOBV while PUUV infected patients did not show this correlation but showed higher IL-12 levels. Exact data on the interval between onset of disease and sampling time point could not be determined, however the authors state that no time-dependence of cytokine expression was seen. Lindgren et al evaluated in a longitudinal study T-cell response in early and late phase of acute PUUV infection during an hantavirus epidemic highlighting the role of CD8+ T cells in the early phase of acute hantavirus infection and describe the induction of inhibitory components in the late phase to limit pro-inflammatory immune response (average day 6 and 10, respectively) [13].

It has been suggested that HFRS pathogenesis is likely to be a complex multifactorial process that includes contributions from immune responses, platelet dysfunction, dysregulation of endothelial cell barrier functions and hosts' genetic factors [16]. Hence, we proposed that disparity of pro- and anti-inflammatory cytokines is responsible in part for the pathology seen in human hantavirus infection. In the present study, we aimed to investigate longitudinally expression of cytokines in the early and late phase during the course of acute infection in a hantavirus epidemic in 2010. We want to analyze timing and intensity of pro- and anti-inflammatory cytokines seen over the course of the infection. In our study we aimed to systematically analyze clinical and immunological characteristics in a cohort of 64 patients hospitalized with severe acute hantavirus infection. Furthermore, we analyzed cytokine expression during the early and late acute phase in comparison to healthy control subjects.

## Results

### Patient characteristics and clinical findings

**Figure 1:**
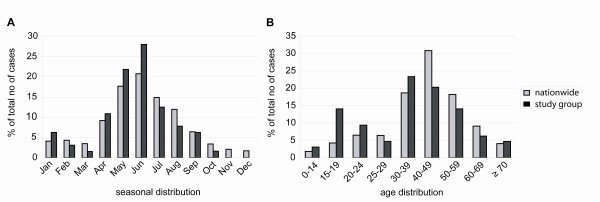
**Comparison of seasonality (A) and age distribution (B) of hantavirus cases in Germany and our study cohort**. Data source on nationwide patient data: Robert Koch Institute.

The patient cohort analyzed in our study consisted of 64 patients (Table 1). 89.7% of patients were adults while 10.3% were children and adolescents. This cohort was representative regarding age distribution and seasonality compared to nationwide data (Figure 1). A similar seasonal distribution was detected in our cohort and for nationwide cases with a peak in June. Age distribution showed a higher percentage of patients in the age group 15-19 years in our cohort compared to nationwide data (14.1% vs. 4.2%, respectively) and the highest proportion of patients in the age group 30-39 years compared to the highest incidence in the age group 40-49 years nationwide, respectively (Figure 1).

**Table 1:** Characteristics of 64 hantavirus-infected patients included in the study.

Patients characteristics (n = 64)	
male	67.2%
female	32.8%
adult	89.7%
children/adolescents (age < 18 years)	10.3%
mean age, years ± SD	38.6 ± 16.4
mean hospitalization time, days ± SD	6.7 ± 4.5
mean time until admission, days ± SD	5.8 ± 3.5

Sex, age, hospitalization time and mean time until admission to hospital are given.

Mean time from onset of disease until admission was 5.8 days, mean hospitalization time was 6.7 days. The most common symptoms reported on admission were fever, lumbalgia, headache, nausea, vomiting and abdominal pain (Table 2). All patients had impaired renal function to some degree on admission with an increase in retention parameters over the course of disease, two patients (both children/adolescents) required intermittent hemodialysis.

**Table 2:** Mean laboratory values on admission and minimum/maximum values during hospitalization of the study group.

parameter	value on admission ± SD	range	reference values
**creatinine on admission, mg/dl**	4.2 ± 2.5	1.0 - 11.7	0.1 - 1.3

**max. creatinine, mg/dl**	5.5 ± 2.7	1.1 - 11.9	0.1 - 1.3

**platelets on admission,/nl**	168 ± 99	38 - 531	150 - 440

**min. platelets,/nl**	143 ± 99	37 - 531	150 - 440

**C-reactive protein on admission, mg/l**	57.1 ± 34.3	9.6 - 147.8	< 5

**max. C-reactive protein, mg/l**	66.0 ± 37.1	10.5- 148.0	< 5

**WBC on admission,/nl**	9.6 ± 3.2	4.7 - 16.4	4 - 10

**max. WBC,/nl**	11.2 ± 3.1	4.7 - 19.9	4 - 10

**AST on admission, U/l**	43.6 ± 25.7	14.0 - 156.0	< 35

**max. AST, U/l**	80.9 ± 120.8	27.0 - 907.0	< 35

**ALT on admission, U/l**	39.9 ± 24.0	10.0 - 113.0	< 35

**max. ALT, U/l**	93.0 ± 105.7	10.0 - 745	< 35

Results for important clinical laboratory markers during hantavirus infection are described.

**Figure 2:**
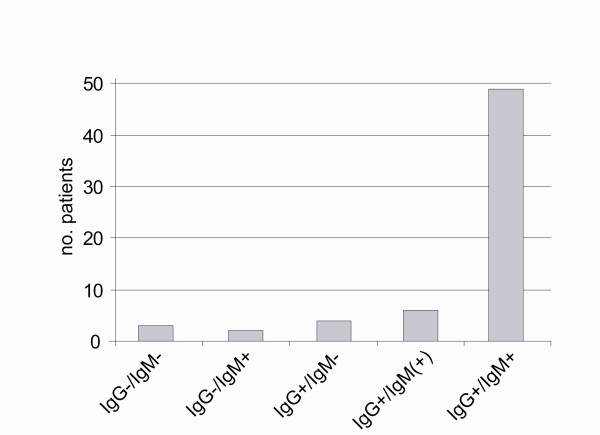
**Detection of anti-*Puumala *virus antibodies (IgG, IgM) in hantavirus-infected patients on hospital admission**. Three patients presented without any detectable anti-PUUV antibodies on admission but seroconversion was detected in follow-up samples.

Laboratory findings on admission were elevated creatinine values with a mean of 4.2 mg/dl, CRP of 57.1 mg/l, WBC of 9.6/nl, AST of 43.6 U/l, ALT of 39.9 U/l and thrombocytopenia of 168/nl (Table 2). During the course of disease, serum creatinine increased to a maximum mean value of 5.5 mg/dl and platelets decreased to a minimum mean value of 143/nl. Urine examination on admission showed proteinuria and hematuria in 92.2% and 75% of all patients, respectively. Pathological findings in chest x-ray were found in 41. 9% and ultrasound findings such as ascites, splenomegaly, hepatomegaly and kidney enlargement were found in 28.6%, 55.8%, 51.2% and 57.4%, respectively (Table 3). Further, several unusual findings were observed in single patients which are not frequently associated with hantavirus infection (Table 3). On admission, the majority of patients were seropositive for anti-PUUV IgG and/or IgM antibodies (Figure 2). However three patients (4.7%) presented without any detectable anti-PUUV antibodies on admission but seroconversion could be demonstrated in follow-up samples.

**Table 3:** Symptoms and clinical findings of hantavirus-infected patients.

Symptoms	Frequency
fever	85.9%
lumbalgia	62.5%
headache	54.7%
nausea	45.6%
vomiting	39.1%
abdominal pain	35.9%
myalgia	28.1%
dizziness	25.0%
bradycardia	26.3%
vision disturbance	20.6%
diarrhea	20.3%
cough	21.9%
arthralgia	16.6%
collaps	10.9%
dyspnoea	12.5%
**Clinical findings**	
proteinuria	59/64 (92.2%)
hematuria	48/64 (75.0%)
pathologic findings in chest x-ray	18/43 (41.9%)
ascites	10/35 (28.6%)
splenomegaly	24/43 (55.8%)
hepatomegaly	22/43 (51.2%)
kidney enlargement	35/61 (57.4%)
**Unusual clinical findings associated with hantavirus infection**	
pulmonary edema	
acute glaucoma attack	
severe neurological involvement	
exanthema	
abortion	
hepatitis	
interstitial nephritis	
seizure	
hypertensive urgency	
Liver vein congestion	

The frequency of clinical symptoms and findings is shown to point out common and uncommon symptoms. Additionally, unusual clinical findings are depicted separately, these were found once in different patients, respectively.

### Cytokine levels in patients with acute hantavirus infection versus healthy controls

**Figure 3:**
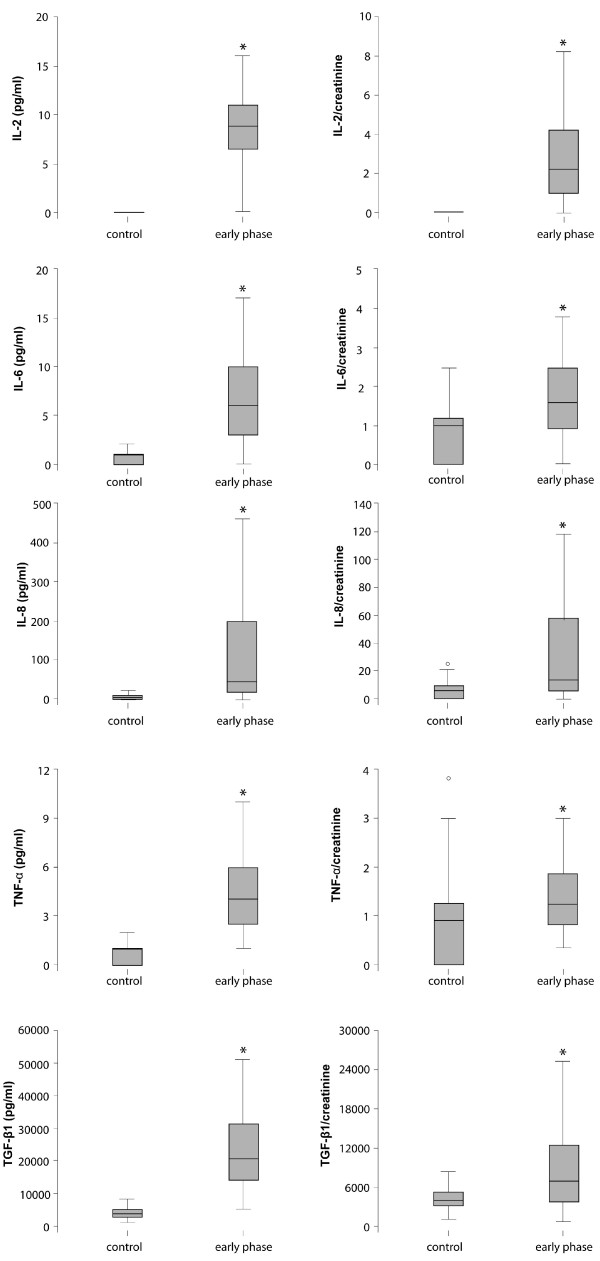
**Cytokine expression in patients (n = 64) with acute hantavirus infection during the early phase of disease (average 6 days after onset of symptoms) versus healthy controls (n = 39)**. The line in the middle of each box represents the median. The lower and the upper edges of the box are the 1st and 3rd quartile, respectively. There was a significant increase of IL-2, IL-6, IL-8, TGF-β1 and TNF-α during early acute hantavirus infection versus healthy controls. To exclude accumulation of cytokines due to impairment of renal excretion function during the early phase of acute disease, cytokine/creatinine ratios from the respective serum creatinine of every sample were calculated. Significant boxplots (p < 0.05) are marked with *.

The early phase of acute hantavirus infection (average 6 days after onset of symptoms) was characterized by significant elevation of cytokine expression of IL-2, IL-6, IL-8, TGF-β1 and TNF-α versus healthy controls (Table 4 and Figure 3). As patients showed strongly impaired kidney function during the early phase of acute disease and cytokines can accumulate in the blood because of impaired renal excretion, additionally cytokine/creatinine ratios were analyzed which corresponded in their significance fully with the blank cytokine expression. Significantly elevated serum levels of IL-2, IL-6, IL-8, TGF-β1 and TNF-α were also observed when the late phase of acute disease was compared with healthy controls, demonstrating a profound and prolonged pro-inflammatory response to hantavirus infection. No significant difference was seen for IFN-γ, IL-10 and IL-5 or their respective cytokine/creatinine ratio compared to healthy controls (Table 4).

**Table 4:** Comparison of cytokines in early and late acute hantavirus infection and healthy controls.

parameter	healthy controls (n = 39)	early phase (n = 64)	early phase* (n = 21)	late phase (n = 21)	p_1_	p_2_	p_3_
**IL-2 (pg/ml)**, **IL-2/creatinine**	2.2±8.6 2.7±12.2	9.5±7.2 3.8±4.5	7.2±5.6 3.2±3.9	14 ±17 15±25	**<0.0001** **<0.0001**	**<0.0001** **<0.0001**	0.08 **0.005**

**IL-5 (pg/ml)** **IL-5/creatinine**	3.3±8.4 3.5±7.8	0.6±2.1 0.3±1.4	0.4±0.6 0.2±0.5	0.3±0.6 0.2±0.5	0.82 1.00	0.63 0.61	0.56 0.65

**IL-6 (pg/ml)** **IL-6/creatinine**	1.1±1.6 1.3±2.0	7.8±9.8 2.5±3.1	8.2±6.1 3.3±3.6	2.6±2.4 1.8±1.2	**<0.0001** **0.0003**	**0.0002** **0.004**	**0.001** 0.17

**IL-8 (pg/ml)** **IL-8/creatinine**	13±32 14±34	563±1809 161±406	444±1076 147±378	130±230 93±163	**<0.0001** **0.0001**	**0.001** **0.002**	0.16 0.57

**IL-10 (pg/ml)** **IL-10/creatinine**	2.4±5.5 2.6±5.7	5.2±8.4 2.2±5.6	8.3±10.7 4.4±8.6	0.7±2.0 0.6±1.9	0.014 0.18	0.17 0.14	**0.001** **0.003**

**TNF-α (pg/ml) TNF-α/creatinine**	0.8±1.0 0.9±1.1	5.2±4.2 1.7±1.4	6.4±4.6 2.2±1.9	3.6±3.1 3.0±2.9	**<0.0001** **0.001**	**<0.0001** **<0.0001**	**0.001** 0.09

**TGF-β (pg/ml)** **TGF-β/creatinine**	4203±2132 4529±2266	23708±12207 11178±13749	21581±9068 8605±7184	32847±13513 29239±18032	**<0.0001** **0.004**	**<0.0001** **<0.0001**	**0.004** **0.0001**

**IFN-γ (pg/ml)** **IFN-γ/creatinine**	0.3±1.8 0.4±2.5	1.1±6.7 0.3±2.2	2.9±11.6 1.0±3.9	10.5±33.5 7.5±23.9	0.41 0.41	0.23 0.23	0.29 0.29

Statistical significant differences between healthy controls, patients with early hantavirus infection and patients with late hantavirus infection are shown in bold.

All data are given as mean±1SD. p1 = early phase acute hantavirus infection (n = 64) vs. healthy controls; p2 = late phase acute hantavirus infection vs. healthy controls; p3 = early phase (n = 21) vs. late phase acute hantavirus infection. P-values were calculated using Mann-Whitney-U and Wilcoxon Signed-Rank tests. Adjustment for multiple testing was done according to the method of Bonferroni. Only p-values of <0.01 after adjustment were considered to be significant and are bold printed. *only early phase samples of patients for which a corresponding late phase sample was available. To exclude cytokine accumulation due to impairment of renal excretion function, cytokine/creatinine ratios were calculated.

### Cytokine levels in early versus late phase of acute hantavirus infection

**Figure 4:**
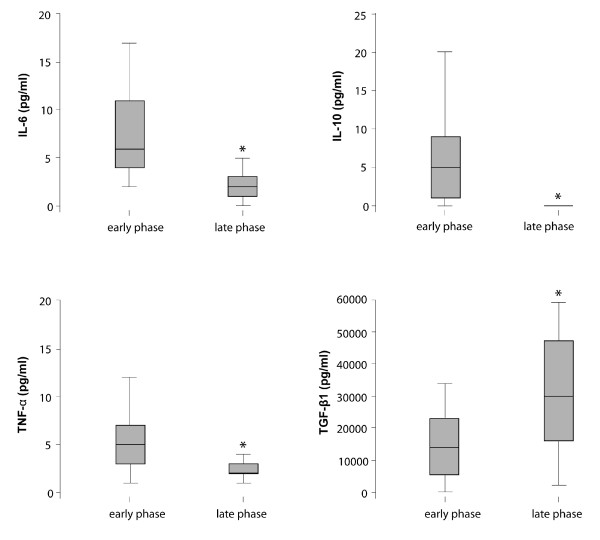
**Cytokine expression during early (average 6 days after onset of symptoms) and late phase (average 12 days after onset of symptoms) of acute hantavirus infection in 21 patients**. The line in the middle of each box represents the median. The lower and the upper edges of the box are the 1st and 3rd quartile, respectively. There was a significant decrease of IL-6, IL-10 and TNF-α and a significant increase of TGF-β1. Significant boxplots (p < 0.05) are marked with *.

To further analyze the relation of pro- and anti-inflammatory cytokines during the course of disease in acute hantavirus infection, we compared expression of IL-2, IL-5, IL-6. IL-8, IL-10, IFN-γ, TNF-α and TGF-β1 in early versus late phase of acute hantavirus infection (average 6 and 12 days after onset of symptoms, respectively) (Table 4 and Figure 4). A significant decrease from early to late phase was seen for IL-6, IL-10 and TNF-α while a significant increase in TGF-β1 was seen. No significant differences were seen for IL-2, IL-5, IL-8 and IFN-γ although there was a trend for increase in IL-2 (Table 4). Rapid decrease of the several main pro-inflammatory mediators as well as increased TGF-β1 during the late phase indicate induction of inhibiting mechanisms limiting early acute phase immune response (Figure 4).

### Correlation of cytokine expression with disease severity

Severity of disease defined by higher deviation from the reference value of main blood parameters creatinine and platelets was associated with high pro-inflammatory cytokines and low TGF-β1 levels (Table 5). High serum creatinine was associated with high IL-6, IL-8 and TNF-α but low TGF-β1 levels. Low blood platelet counts were associated with high IL-6, IL-10 and TNF-α and low TGF-β1. In addition, time interval from beginning of symptoms until measurement of cytokines was associated with high TGF-β1. These findings suggest increasing serum TGF-β1 and an association of high TGF-β1 with improving kidney function and normalizing platelet counts in the late phase of disease. When correlating TGF-β1 with pro-inflammatory cytokines, TGF-β1 was associated with low IL-8, IL-10 and TNF-α levels. Although high CRP was associated with high IL-6, IL-8 and TNF-α, it did not correlate with TGF-β1.

**Table 5:** Correlation analysis of cytokine expression for creatinine/cytokine ratio, platelet/cytokine ratio and CRP/cytokine ratio.

parameter	r	p
**Creatinine/IL-6**	0.337	0.005

**Creatinine/IL-8**	0.316	0.009

**Creatinine/TNF-α**	0.560	<0.001

**Creatinine/TGF-β1**	-0.265	0.029



**Platelets/IL-6**	-0.396	0.001

**Platelets/IL-10**	-0.367	0.002

**Platelets/TNF-α**	-0.391	0.003

**Platelets/TGF-β1**	0.421	<0.001



**TGF-ß1/IL-8**	- 0.26	0.030

**TGF-ß1/IL-10**	- 0.42	<0.0001

**TGF-ß1/TNF-α**	- 0.30	0.017



**CRP/IL-6**	0.49	<0.0001

**CRP/IL-8**	0.34	0.004

**CRP/TNF-α**	0.46	0.0001

## Discussion

Southwest Germany is a main endemic area for PUUV infection with a strong increase in hantavirus infections in 2010 [5,17]. Most studies on PUUV so far have described the clinical setting of NE in Western or Northern Europe and the Balkans [4,7,18,19], but large patient numbers have rarely been described from Germany [20,21]. A recent study retrospectively describing 75 hantavirus infected patients over a 8-year period in Southwest Germany found comparable clinical characteristics, however, with average lower creatinine and lower platelet numbers than observed in our study [21]. It should be noted, that lower creatinine values are not expected to be associated with lower platelet numbers in cases of hantavirus infection. Hospitalization time was longer with average 9 days while our cohort had an average mean hospitalization time of 7 days. Interestingly, we observed a higher proportion of organ pathologies of spleen, liver and lung with approx. 56%, 51% and 41% compared to 50%, 13% and 16%, respectively. We want to highlight especially findings of pulmonary involvement as there is evidence that the strict differentiation regarding kidney and lung pathology in Old and New World hantaviruses is not useful when studying pathogenesis, further, pulmonary findings in Old Wold hantavirus infection are increasingly recognized [22-25]. In contrast to nationwide data and other studies, we observed a higher proportion of young patients aged 15-19 years, however most studies are undertaken from medical disciplines focusing on adult patients and access to a large pediatric unit in our study site might bias these findings. We could further confirm the extreme heterogeneity of NE mimicking a range of other diseases especially in the initial phase of disease and found a wide spectrum of unusual findings and complications in single patients during the course of disease in patients of the 2010 epidemic. Some of these findings might be coincidental, however they are complicating diagnosis. It is not clear if divergent clinical findings to other studies such as higher number in organ pathology or higher patient numbers in the young age group are hints for a changing disease manifestation of hantavirus infection in the 2010 epidemic or biased by analyzing a subgroup with severe disease leading to hospitalization at a large University hospital and intensive clinical monitoring. So far, there are no other studies published on the 2010 hantavirus epidemic in Germany.

Diagnosis of hantavirus infection is confirmed by serological assays using the immunodominant nucleocapsid protein antigen [26-28]. By the time patients are symptomatic, most patients develop IgM and IgG antibodies [26,27,29]. Only few cases show delayed IgM response, while the IgG response is delayed in 10% of acute-phase IgM-positive sera [30,31]. For early testing or confirmation of questionable serological results, viral RNA can be detected by RT-PCR, but is not routinely applied [30,32]. Although most patients in our study group presented with positive anti-PUUV IgG and IgM antibodies, a subset of patients were seronegative, had only borderline IgM or IgG antibodies on admission but seroconverted during the course of disease. In contrast, in a recent report by Schilling et al., most patients presented with IgG and all patients presented with IgM antibodies during the acute phase in a PUUV epidemic in 2004 in Southern Germany [30]. These findings highlight that antibody testing at multiple time points is conclusive if the clinical picture is suspicious for acute hantavirus infection. In case of atypical presentation, molecular diagnostic methods such as PCR and sequencing of the infecting strain should be considered [30,32].

Many studies have shown so far that cytokines play an important role in the pathogenesis of human hantavirus infection in both HFRS and HPS [14,33-36]. One of the most investigated cytokine in acute hantavirus infection of both syndromes HFRS and HPS is TNF-α which was almost uniquely found overexpressed in acute hantavirus infection [11,14,37]. TNF-α is produced by monocytes, macrophages and T cells and is an inducer of NO synthase with important effects on capillary endothelial permeability.

Experimental data from an endothelial cell culture model showed that *Hantaan *virus which causes HFRS in Asia induces TLR 4, responsible for enhanced production of TNF-α as well as IFN-β1 and IL-6 [34]. In two clinical studies in Belgium and Brazil, polymorphisms in the TNF-α promoter were found associated with a more severe course of disease in PUUV infection and susceptibility to HPS, respectively [11,38]. A pauci-symptomatic case of PUUV infection in a patient under anti TNF-α treatment supports a crucial role of TNF-α in hantavirus pathogenesis [39].

One of the main pro-inflammatory proteins beside TNF-α is IL-6 which is also involved in induction of acute inflammatory responses. Severity of disease in HFRS is correlated with serum and urine levels of IL-6 [40,41]. Outinen et al. found that high IL-6 is associated with disease severity and increased hospitalization time of PUUV infected patients [33]. Associations of high serum creatinine and low platelet counts with serum levels of TNF-α and IL-6 in our patients support the role of these cytokines in HFRS. Further, in fatal cases with HPS, high numbers of cytokine-producing cells (IL-6, TNF-α, IFN-γ and IL-2 among others) were seen in the lung and spleen tissues of HPS patients, indicating a role for cytokines also in the target organ [10]. In our study we could show a significant decrease of several main inflammatory cytokines from early to late phase acute disease and induction of an immunosuppressive mechanism characterized by significant increase in TGF-β1 which is supposed to have a negative influence on lymphocyte activation [42]. In contrast to others we did not see an increase in IL-10 during acute infection but only a significant decrease of IL-10 from early to late phase whereas increased TNF-β1 was seen in both early and late phase versus healthy controls and additionally showed a significant increase from early to late phase samples [14,16,43]. These differences in the detection of certain cytokines could be due to the varying sampling time points after onset of disease or a mixture of patients at early and late phase of acute disease in studies which did not evaluate the time span from onset of first symptoms. Near-normal platelet numbers as well as only a small increase from creatinine on admission to maximum creatinine value indicated that - similar to most other studies- we missed the very early phase of disease in our cohort as well. Generally, due to the unspecific nature of hantavirus symptoms, it is very difficult to evaluate the very early cytokine response as patients are rarely seen and diagnosed within the first days of infection [15].

In general, our findings support the induction of an inhibitory effect from early to late course of disease which limits early acute phase pro-inflammatory response. Interestingly, this immunoregulatory effect seems to be optimized for virus persistence in the reservoir host characterized by asymptomatic infection: Easterbrook et al. found regulatory T cells mediating Seoul virus persistence in rats, possibly through elevated transcription and synthesis of TGF-β1 and suppression of TNF in the natural reservoir host [44]. In deer mice persistently infected with Sin Nombre virus, T cells from acutely infected mice synthesized a broad spectrum of cytokines, including IFN-γ, IL-4, IL-5, and TGF-β1, but not TNF, lymphotoxin, or IL-17. However, in T cells from persistently infected deer mice, only TGF-β1 was expressed by all cell lines, whereas some cell lines expressed reduced levels of IFN-γ or IL-5 [45].

## Conclusions

The strong association of high TGF-β1 serum level with low serum creatinine and high platelet counts in our study suggests a protective immunoregulatory role of high TGF-β1 mitigating clinical symptoms in the late acute phase of hantavirus-infected patients. High TGF-β1 serum levels inhibit pro-inflammatory cytokines and prevent the patient from further cytokine-induced inflammations and injuries of tissues and organs. Strong associations of high TGF-β1 with low IL-8, IL-10 and TNF-α serum levels during acute hantavirus infection support this hypothesis. The strong association of high TGF-β1 with high platelet counts point to platelets as an additional source of increased serum TGF-β1 during convalescence of the patients. Thus, the strong inflammatory immune response induces a counter regulation with increased TGF-β1 originating from activated immune cells such as T lymphocytes, monocytes and macrophages. The counter regulation increased the number of platelets to normal platelet counts and these platelets might be activated and release additional TGF-β1. The observation of decreased TGF-β1 serum levels in patients with hantavirus pulmonary symptoms are in line with our observation of high TGF-β1 serum levels in asymptomatic convalescent patients [40].

## Methods

### Study population

Sixty-four hospitalised patients diagnosed with acute hantavirus infection at the University Hospital Heidelberg were analyzed for clinical characteristics by hospital chart. Hantavirus antibodies and cytokine levels were determined in serum samples obtained during clinical routine. Early phase samples of acute disease were from the first presentation upon admission to the hospital, on average 6 days after onset of symptoms. An additional late phase sample was available for 21 of those patients, on average 12 days after onset of symptoms. All samples were analysed for cytokine expression levels. Thirty-nine healthy blood donors (age 37.2±12.2 years) served as controls. Data on nationwide demographic characteristics of hantavirus cases were obtained from the central federal institution for disease control Robert Koch Institute (SurvStat@RKI, http://www3.rki.de/SurvStat, data status: 14.02.2011).

### Detection of hantavirus antibodies

Sera were tested for IgG and IgM antibodies using the *recom*Line hantavirus assay (Mikrogen, Munich, Germany), coated with specific hantavirus nucleocapsid antigens of *Puumala *virus, *Hantaan *virus and *Seoul *virus. Patients were included in the study if both IgG and IgM for *Puumala *were positive on admission or during the course of disease.

### Determination of serum cytokines

IL-2, IL-5, IL-6, IL-8, IL-10, IFN-γ, TGF- β1 and TNF-α were measured by ELISA (R&D Systems, Wiesbaden, Germany) in 64 early and 21 late phase samples.

To exclude cytokine accumulation due to dysfunction of renal excretion function in our analysis, in addition, cytokine/creatinine ratios were calculated.

### Statistical analysis

Mann-Whitney-U, Wilcoxon signed rank and Spearman correlation coefficient test were applied using the Statistical Package for the Social Sciences (SPSS, Chicago, USA). Adjustment for multiple testing was done according to the method of Bonferroni. P values of <0.05 after adjustment were considered significant.

### Ethical approval

This study was approved by the Ethics Committee of the Faculty of Medicine, University Heidelberg. Written consent was obtained from patients and healthy controls.

## Authors' contributions

MS and VD carried out the immunoassays for cytokine analysis. MS performed the statistical analysis. IE and PS developed the design of the study, IE coordinated the study and collected clinical data of the patients. PS and UB performed the immunoassays for hantavirus antibodies. VD, GO and PS drafted the manuscript. All authors read and approved the final manuscript.
